# Male Genital Morphology and Its Influence on Female Mating Preferences and Paternity Success in Guppies

**DOI:** 10.1371/journal.pone.0022329

**Published:** 2011-07-22

**Authors:** Clelia Gasparini, Andrea Pilastro, Jonathan P. Evans

**Affiliations:** 1 Department of Biology, University of Padova, Padova, Italy; 2 Centre for Evolutionary Biology, School of Animal Biology, University of Western Australia, Crawley, Australia; University of Jyväskylä, Finland

## Abstract

In internally fertilizing species male genitalia often show a higher degree of elaboration than required for simply transferring sperm to females. Among the hypotheses proposed to explain such diversity, sexual selection has received the most empirical support, with studies revealing that genital morphology can be targeted by both pre-and postcopulatory sexual selection. Until now, most studies have focused on these two episodes of selection independently. Here, we take an alternative approach by considering both components simultaneously in the livebearing fish, *Poecilia reticulata*. We allowed females to mate successively (and cooperatively) with two males and determined whether male genital length influenced the female's propensity to mate with a male (precopulatory selection, via female choice) and whether male genital size and shape predicted the relative paternity share of subsequent broods (postcopulatory selection, via sperm competition/cryptic female choice). We found no evidence that either episode of sexual selection targets male genital size or shape. These findings, in conjunction with our recent work exposing a role of genital morphology in mediating unsolicited (forced) matings in guppies, further supports our prior speculation that sexual conflict may be an important broker of genital evolution in this species.

## Introduction

In animals with internal fertilization, male genitalia typically exhibit extreme morphological divergence, even among closely related species. Understanding the evolutionary basis for this variation has been a key goal in evolutionary biology [1]–[3]. Among the hypotheses proposed to explain such extraordinary patterns of divergence, the sexual selection hypothesis has gained the most empirical support (see [1], [3]–[4]). However, the mechanisms of sexual selection responsible for this diversity remain largely unclear.

The sexual selection hypothesis predicts that the size and shape of genital traits evolve in response to selection for increased reproductive success via several non-mutually exclusive mechanisms [1], [5]. Although the majority of studies examining genital evolution via sexual selection emphasise the role of postmating mechanisms, such as sperm competition and cryptic female choice (e.g. see [1], [3], [6]), other studies have revealed a role for premating mechanisms of sexual selection, and in particular for female choice favouring longer external genitalia [7]–[10]. Surprisingly, however, these two components of pre- and postcopulatory sexual selection have rarely been incorporated within a single study.

The guppy, *Poecilia reticulata*, is a freshwater fish with internal fertilization and a promiscuous mating system in which males alternate between courtship and forced matings to achieve copulation. Males inseminate females using a modified anal fin that functions as an intromittent organ (the gonopodium), a structure that exhibits considerable variability in size and shape both within and among populations [11]–[12]. Indirect evidence from guppies and other poeciliid fishes suggest the length of the male's gonopodium is subject to sexual selection through precopulatory female choice [7], [9], [13], although a recent study has also revealed that gonopodial traits are associated with the success of forced matings [12]. However, despite indirect evidence that male genital size and shape differs among populations according to the level of sperm competition [11]–[12], we have yet to determine whether postcopulatory sexual selection explicitly targets these traits, and the extent to which pre- and postcopulatory sexual selection work together to favour specific genital traits.

In this study, we test whether the size and shape of the male's gonopodium is associated with precopulatory (i.e. mating success) and postcopulatory success (i.e. paternity success) following two successive solicited copulations in guppies. We conducted mating trials in which females were allowed to mate successively with two males. During these trials we used latency to mate as a proxy for female mating preferences (precopulatory success) and paternity analyses to estimate relative fertilization rates (postcopulatory success). We then used geometric morphometric analyses to describe male genital morphology, and linear measurements of the gonopodium to estimate genital length. Our subsequent analyses related male genital shape and length to mating preferences and fertilization success.

## Materials and Methods

### Ethics statement

This study was approved by the University of Western Australia's Animal Ethics Committee (Research Integrity Office, permit number 05/100/513).

### Study population and its maintenance

All animals used in this experiment were laboratory born descendents of wild-caught fish collected from Alligator Creek (30 km south of Townsville) in Queensland, Australia. Fish were maintained at a constant temperature of 26±1°C under a 12 h∶12 h day-night cycle and fed live brine shrimp and commercial flake twice daily. Virgin females were used for the experiments to ensure that females were sexually receptive during the mating trials [14] and to ensure that sperm stored from prior males did not contribute towards the broods genotyped for paternity analyses (see below). To raise virgins, juvenile females were separated from their male brood mates as soon as their sex could be determined (*ca.* 4 weeks-old) and then reared in single-sex tanks until used in the mating trials.

### Mating trials

Experimental males were taken from stock aquaria and isolated from females for at least 3 days to ensure that they entered the mating trials with fully replenished sperm stores [15]. On the evening before each mating trial, a sexually mature virgin female (approx. 18 month-old) was placed individually into the mating tank (43×23×25 high, filled to a depth of 21 cm) and left to settle overnight. On the following morning, an experimental male was placed in the mating arena and allowed to copulate once with the female. We measured ‘copulation latency’ (time to mate) in these ‘no-choice’ trials as an estimate of female preferences [16]. We also counted the total number of courtship displays (termed sigmoid displays) performed by the male, as this trait has been found to be correlated with both pre- and postmating success in this species [14], [17]. As soon as the focal pair successfully copulated, we removed the first male for subsequent morphological analyses (see below) and left the female alone for 5 minutes before introducing the second male to the tank. We then scored copulation latency and courtship displays as before. As soon as the second male had successfully mated with the female, we removed the male for morphological analyses. If a male did not copulate or exhibit any sexual behaviour within 10 minutes, we removed it from the experimental tank and replaced him with a different male. In total, we carried out 41 successful double-mating trials (*n* males = 82). After each trial had finished, the female was placed individually into a 2 L container until she gave birth. At this point, fin clips taken from the mother and whole bodies of the offspring were preserved in absolute ethanol for subsequent paternity analysis. Twenty of the 41 double-mated females gave birth to broods.

### Male genital morphology

After successfully copulating with the female, males were euthanized and immediately photographed using a digital camera (Nikon CoolPix 5400). A ruler was included in each photograph to calibrate the subsequent measurements. Digital photos were analysed using image analysis software (UTHSCSA Image Tool v3.0, http://ddsdx.uthsca.edu/dig/download.html). For each male, we estimated standard length (SL), gonopodium length, and the area of the male's body covered by orange spots. A fin clip was taken at this point and preserved in absolute ethanol for subsequent DNA extraction. The remainder of each male's body was then preserved in Dietrich's fixative (30% pure ethanol, 10% formalin, 2% glacial acetic acid, 58% H_2_O). A digital image of each male's gonopodium was subsequently captured using a Leica DFC320 fitted to a Leica MZ75 stereomicroscope under transmitted light and dark field illumination. The image was captured at ×50 magnification and focused on the distal tip of the gonopodium, which is the portion of the intromittent organ that physically contacts the female genital tract during copulation (see also [12], [18]–[19]).

**Figure 1:**
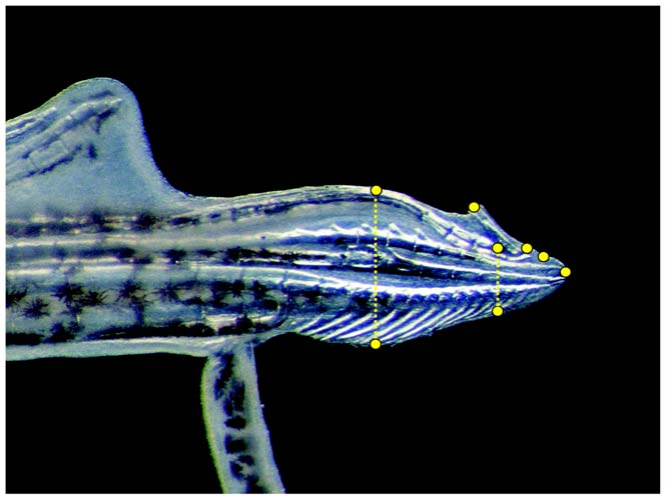
Photograph of the distal tip of the gonopodium of *Poecilia reticulata* in lateral view. Eight landmarks (yellow dots) were superimposed on each image using geometric morphometric software (see text for details).

We estimated variation in the shape of gonopodium's distal tip using geometric morphometric analyses (reviewed in [20]) following methods described in Evans et al. [12]. Eight fixed landmarks were superimposed at homologous points on each image (see fig. 1). Landmarks were digitized using tpsDig2 software [21], and for each male, landmark data were analysed using tpsRelw v1.42 software [22]. This generated relative warp scores, which describe shape variation as deviations from a consensus shape. Relative warp scores were subject to relative warp analysis, which corresponds to a principal components analysis and serves to reduce multivariate shape data to relative warps (RWS) that describe most of the variation in shape. The relative warp analyses returned 4 relative warps, explaining >80% of the variance in gonopodia's tip shape (hereafter referred to as RWS-1–4).

### Paternity analysis

Genomic DNA was extracted from newborn offspring using the Chelex protocol [23] and from adults using a standard salting out protocol [24]. We then used up to five microsatellite markers to assign paternity, including TTA, Pr39, Pr67, KonD15, KonD21 (Genbank accession numbers: AF164205, AF467903, AF533589, AF368429, AF368430). PCR amplifications were performed following the methods outlined in Gasparini et al. [25] on a GeneAmp PCR System 2700 Thermocycler (Applied Biosystems, CA, USA). Amplified fragments were separated by electrophoresis on an ABI 3100 sequencer (ABI PRISM, Applied Biosystems), using 400 HD ROX (Perkin-Elmer, Applied Biosystems) as a size standard (http://www.bmr-genomics.com). PCR products were visualized using GeneMarker V. 1.91 (http://www.softgenetics.com) and paternity was assigned to offspring according to allele sharing between putative sires, mother and offspring.

### Statistical analyses

Data were checked for normality before analyses, and data for copulation latency (our estimate of female mating preferences) were square root transformed to achieve normality. All means are reported with their standard errors. We ran separate models to test influences of gonopodial traits on latency (precopulatory success) and on paternity share (postcopulatory success). For copulation latency (latency to mate with the second male was the dependent variable) we ran two models, one including only gonopodium length as a predictor and the second including both gonopodium length *and* the other precopulatory variables (see below). To analyse paternity data, we ran one model including gonopodium length and shape as predictors, and one including both gonopodial traits and precopulatory traits. The rationale for this choice is that we wanted to see first whether gonopodium size and shape affected precopulatory preference and paternity success, and then to control for the confounding effects of other precopulatory male traits. For copulation latency, we included only genital length as a predictor, as there was no *a priori* reason to expect the shape of gonopodium's tip to affect attractiveness. Predictor variables for both latency (premating success) and paternity analysis (postmating success) consisted of differences in trait values between the two males (2^nd^ male trait–1^st^ male trait) in gonopodium length and shape, body length (SL), courtship rate and orange coloration. Courtship rate refers to the number of displays per minute. We used G-Power [26] to determine the statistical power of our tests on paternity share (simplified to paired t-tests). We calculated the power to detect a significant effect at α = 0.05 (two tailed) given a medium effect (*sensu* Cohen [27]). Statistical analyses were performed using SPSS v. 18.0 (SPSS Inc, Chicago, IL, USA) and GenStat v. 12 (VSN International Ltd, Hemel Hempstead, UK). Postcopulatory success was examined using paternity share of the second male to mate (P_B_) and showed a binomial distribution and overdispersion (overdispersion parameters for model c and d were ϕ = 0.991 and ϕ = 1.049, respectively), similar to previous studies (see [17]). Overdispersion was corrected using the Williams procedure [28], implemented in GenStat (EXTRABIN command). All probabilities are two-tailed.

## Results

### Mating success

Descriptive statistics for male traits are reported in table 1 (a, b). In our mating trials females mated more quickly with the second male in 28 cases out of 41 (68.3%, sign test, *P* = 0.028). Copulation latency was not related to differences in gonopodial length between the two males (see table 2a). When we included the other precopulatory variables in the model, the only significant predictor was the differences in courtship rate performed by the males, with mating success favouring males with relatively high courtship rates (*P* = 0.001, see table 2b).

**Table 1:** Descriptive statistics for male traits used.

Precopulatory success (copulation latency)				Postcopulatory success (paternity share)			
	Mean first male (SD)	Mean second male (SD)	Mean differences (min–max)		Mean first male (SD)	Mean second male (SD)	Mean differences (min–max)
**(a)**				**(c)**			
gonopodium length (mm)	3.46 (0.30)	3.50 (0.39)	0.04 (−0.64–0.87)	gonopodium length (mm)	3.43 (0.29)	3.46 (0.38)	0.03 (−0.49–0.82)
				RWS1	−0.001 (0.05)	−0.01 (0.05)	0.00 (−0.11–0.12)
				RWS2	0.00 (0.03)	0.00 (0.03)	0.00 (−0.07–0.08)
				RWS3	−0.01 (0.02)	0.00 (0.03)	0.01 (−0.07–0.07)
				RWS4	−0.01 (0.02)	0.00 (0.02)	0.01 (−0.06–0.10)
**(b)**				**(d)**			
gonopodium length (mm)	3.46 (0.30)	3.50 (0.39)	0.04 (−0.64–0.87)	gonopodium length (mm)	3.43 (0.29)	3.46 (0.38)	0.03 (−0.49–0.82)
body size (mm)	17.40 (0.85)	17.64 (1.13)	0.24 (−2.68–4.59)	body size (mm)	17.13 (0.59)	17.51 (0.89)	0.38 (−1.44–2.39)
courtship rate	2.77 (6.33)	4.56 (8.66)	1.79 (−29.25–28.70)	courtship rate	3.04 (6.48)	4.83 (8.96)	0.25 (−6.00–9.83)
orange coloration (mm^2^)	8.32 (4.35)	7.88 (4.25)	−0.44 (−10.22–15.01)	orange coloration (mm^2^)	7.37 (3.33)	6.76 (3.59)	−0.61 (−8.17–15.01)

Descriptive statistics for male traits considered in the experiment, including means and their standard deviations (in parentheses) for the first and the second male, and the means of the differences and their ranges between the two competitor males (see ‘Statistical analyses’ section for details). (a) and (b) refer to the precopulatory success (n = 41 pairs, n = 82 males in total); (c) and (d) refer to the paternity share (n = 20 pairs, n = 40 males).

**Table 2:** Results of regression analysis of pre- and postcopulatory success.

Precopulatory success (copulation latency)				Postcopulatory success (paternity share)			
	F	*P*	*b* ± SE		*F*	*P*	*b* ± SE
**(a)**				**(c)**			
gonopodium length	0.216	0.645	−1.448±3.11	gonopodium length	0.52	0.604	1.12±2.16
				RWS1	1.01	0.313	−11.6±11.5
				RWS2	1.11	0.267	−22.0±19.8
				RWS3	1.06	0.288	−26.1±24.5
				RWS4	1.78	0.076	−44.0±24.8
**(b)**				**(d)**			
gonopodium length	0.016	0.901	−0.368±2.95	gonopodium length	0.43	0.670	−0.86±2.01
body size	0.984	0.328	−0.825±0.83	body size	0.82	0.413	0.611±0.746
courtship rate	12.72	0.001	−0.345±0.97	courtship rate	0.64	0.525	−0.137±0.216
orange coloration	2.116	0.154	−0.254±0.17	orange coloration	1.28	0.201	0.159±0.124

Results of linear regression analysis (copulation latency, n = 41, model a,b) and logistic regression analysis (paternity share, n = 20, model c,d). Independent variables (predictors) were differences in traits between the two competing males (see ‘Statistical analyses’ section for details).

### Postcopulatory success

We obtained 390 offspring from 20 double mated females (mean brood size = 19.5±2.2 SE; range = 4–35) and genotyped a total of 450 individuals (60 adults and 390 offspring). Given our sample size of 40 males (20 pairs) our statistical power to detect a significant effect is ∼0.87. Out of 390 newborns genotyped, we were able to unambiguously assign parentage to 346 individuals (>88%). Paternity share was biased towards the second male (in 15 out of 20 families the second male sired a larger proportion of offspring, binomial test *P* = 0.032), with a mean paternity of the second male of 0.73±0.09 (range = 0–1). Descriptive statistics for male traits are reported in table 1 (c,d). As reported in table 2, none of gonopodial traits or other precopulatory variables significantly predicted competitive fertilization rates (see table 2c–d).

## Discussion

The aim of this paper was to determine whether male genital traits influence pre- and postcopulatory success during consensual matings in guppies. Unlike previous work, we found no evidence that male genital size influences mating success. For example, Brooks and Caithness [7] showed that gonopodium length positively influenced the female's ‘orient response’ – which was used as a measure of male sexual attractiveness (see also [16]). There are several possible reasons that may account for the discrepancy in results between the two studies. The populations used were different, and in *P. reticulata* it is known that the target of female preference varies among different populations [29], and therefore preference for gonopodium size could vary as well. In addition, the two studies differ in the experimental set up (males encountered sequentially by a single female vs. several males and females freely interacting at the same time). Importantly, we used virgin females (which can be less choosy than experienced females [14]), which differs from Brooks and Caithness's study and two others on *Gambusia* that used non-virgin females and reported precopulatory preferences for longer gonopodia [9], [13]. Nevertheless, further studies are needed to clarify the role of genital morphology on premating success, using non virgin females and ideally using playback experiments in which males presented to the female differ only in genital length (see for example [9]). Although we were interested in examining the role of male genitalia on mating success, we also considered precopulatory traits in our analysis, including the area of orange coloration and courtship rate – two traits that are known to influence mating success in other guppy populations ([14] and references therein). We found that latency to mate (inversely correlated with female preference) was negatively associated with courtship rate (see also [30]–[31]), but surprisingly not with orange colouration [32].

Our molecular analysis revealed that paternity was biased towards the second of two males to mate with a female, which has also been observed in other studies of *P. reticulata* that employed similar double-mating designs [17], [32]. However, our data did not reveal any association between gonopodium traits (both length and shape of the distal tip) and postcopulatory success. Significant associations between male genital morphology and paternity success have been reported in insects, but to our knowledge the present study is the first to test for this association in vertebrates. For example, in praying mantids, *Ciulfina klassi*, genital shape is correlated to sperm transfer [33], and in the dung beetle *Onthophagus taurus* genital morphology predicted relative paternity share when two males competed to fertilize the eggs from a single female [34].

Our data therefore lend no support for the idea that postcopulatory sexual selection shapes male genital morphology, at least when considering solicited copulations. However, our recent intraspecific comparative work on natural guppy populations suggests that male genital morphology may play a role in mediating the success of unsolicited copulations [12]. In that study we found that the size and shape of the distal tip of the gonopodium (as measured in the present study) were significant predictors of genital contacts and sperm transferred during forced copulations [12], leading us to speculate that postcopulatory sexual selection, mediated by sexual conflict, may explain differences in male genital shape in populations that differ in the level of forced matings. Indeed, these previous findings support a general pattern reported among poeciliid fishes in which males in species that possess relatively longer gonopodia tend to rely more (or exclusively) on forced copulations [35]–[36]. Thus, postcopulatory sexual selection may yet explain variation in male genital traits (both intra- and interspecific variation), but these effects may be manifested through the influence of male genital shape on the success of forced matings, not consensual matings as measured here. We have yet to determine whether male genital morphology predicts paternity success following successive forced matings to test this idea vigorously, although we note that obtaining such data presents a special challenge as ‘successful’ forced copulations are hard to obtain.

In summary, our findings provide no evidence that either pre- or postcopulatory sexual selection shape male genital morphology in the population used in this study. Taken in conjunction with our recent work [12], however, we suggest that sexual conflict may be a more potent broker of sexual selection on genital morphology in this species. Further studies, ideally manipulative (e.g. [37]), are needed to understand how variation in male genital shape mediates sperm transfer during forced copulations, and to confirm the lack of influence on gonopodium size on female premating choice during consensual matings.
